# Factors behind poor cognitive outcome following a thalamic stroke

**DOI:** 10.1007/s00415-024-12777-4

**Published:** 2025-01-07

**Authors:** Julie P. C. Vidal, Lola Danet, Germain Arribarat, Jérémie Pariente, Patrice Péran, Jean-François Albucher, Emmanuel J. Barbeau

**Affiliations:** 1 Brain and Cognition Research Center, (CerCo-UMR 5549), CNRS, University of Toulouse, Toulouse, France; 2 Toulouse Neuroimaging Center (Tonic-UMR 1214), Inserm, University of Toulouse, Toulouse, France; 3 Neurology Department, CHU Toulouse Purpan, Toulouse, France

**Keywords:** Thalamus, Stroke, Cognition, MRI, Outcomes

## Abstract

**Background:**

Thalamic strokes produce neurological, cognitive, and behavioral symptoms depending on the thalamic nuclei involved. While traditionally associated with severe cognitive deficits, recent studies suggest more modest impairments. This study aims to identify the factors that influence the severity of cognitive impairment following thalamic stroke.

**Methods:**

We recruited 40 patients (mean age 51.1) with chronic isolated thalamic stroke and 45 healthy subjects (mean age 48.5) who underwent neuroimaging and neuropsychological assessment. Cluster and principal component analyses were used to discriminate patients from healthy subjects based on cognitive tasks. Disconnectome maps and cortical thickness were analyzed to understand the distant impact of thalamic strokes.

**Results:**

Two cognitive profiles emerged from the cluster analysis. Cluster 1 included mostly healthy subjects (*n* = 43) and patients with no or minor deficits (*n* = 20). Cluster 2 included patients (*n* = 19) and two healthy subjects with severe deficits in verbal memory, executive functions, and attention. Cluster 1 encompassed all patients with right thalamic stroke, while Cluster 2 included all patients with bilateral stroke or mammillothalamic tract interruption. Patients with left-sided stroke were equally divided between clusters. Significant differences between clusters included age, education, interthalamic adhesion disruption, lesion volume, and location. Patients with left-sided stroke in Cluster 2 had more lateral thalamic lesions and greater disruption of the anterior thalamic projection.

**Conclusions:**

Contrary to common expectations, our findings suggest that many patients with thalamic stroke have relatively good cognitive outcomes. In contrast, we identified the factors behind poor outcomes that will help clinicians.

**Supplementary Information:**

The online version contains supplementary material available at 10.1007/s00415-024-12777-4.

## Introduction

Given its high vascularity [1], the thalamus is vulnerable to stroke, resulting in diverse neurological, cognitive, and behavioral manifestations depending on the vascular territory and thalamic nuclei involved [2]. Historically, understanding of thalamic lesions has been based on case studies, linking them to severe cognitive deficits, such as amnesia [3], aphasia [4], spatial hemineglect [5], homonymous lateral hemianopia [6] and auditory agnosia [7], among others.

However, recent publications demonstrated the opposite pattern. Indeed, Liebermann et al. [8] studied a group of 68 patients with an isolated thalamic stroke and mainly demonstrated the absence of cognitive differences with the group of healthy subjects. In another study, the same authors investigated 19 patients, 58% of whom did not exhibit major executive deficits [9]. Similarly, during visual anterograde memory tests, either moderate or a lack of memory deficits were observed in 75% of 17 patients with chronic left, right, or bilateral thalamic lesions [10]. These findings were corroborated by other studies [11, 12]. Finally, a longitudinal study was carried out on 37 patients with an isolated thalamic stroke from the acute to the chronic phase and revealed that patients recovered, at the group scale, no matter which cognitive domain and vascular territory were affected [13].

The discrepancy between earlier and recent research may be due to several factors, including the advent of stroke units allowing the inclusion of patients with subtler deficits in research programs. In addition, the development of high-field MRI neuroimaging helps to determine the exact extent of lesions that may have been underappreciated in earlier studies. Positive bias in earlier cases may have led to reporting more compelling cases rather than patients with modest deficits. Finally, the thalamus consists of gray matter and white bundles, such as the mammillothalamic tract (MTT involved in the Papez’s circuit, [2]) and the interthalamic adhesion (IA, [14]). Lack of investigation into the disruption of these bundles in thalamic lesions may hinder the accurate interpretation of cognitive deficits.

These recent group studies question our understanding of the thalamus’s role in cognition and suggest subgroups of stroke patients, some recovering well and others retaining significant cognitive deficits. Our study aims to determine what factors influence good or poor cognitive outcomes following an isolated thalamic stroke.

## Materials and methods

### Participants

This cohort study included 45 healthy subjects (ages 20–69, mean 48.5, 20 males) and 40 patients (ages 23–75, mean 51.1, 25 males) with ischemic thalamic lesions (left = 28, right = 6, bilateral = 6). Patients were recruited prospectively across two studies from the stroke units of Toulouse hospitals between 2012–2013 and 2019–2021. The first study, approved by the Institutional Review Board “Comité de Protection des Personnes Sud-Ouest et Outre-Mer no. 2–11-0,” included 20 healthy subjects and 20 patients under the age of 80 who had experienced a thalamic stroke. Three marginal extra-thalamic lesions were accepted in this study [15]. The “Comité de Protection des Personnes Ile-de-France IV” authorized the second study. It included 20 healthy subjects and 20 patients under the age of 70 with at least one stroke lesion in the dorsomedian nucleus and no extra-thalamic damage. All Fazekas and Schmidt scores were ≤ 2. Recruitment criteria for both studies included detecting a first symptomatic thalamic infarct regardless of prior complaints or neurobehavioral reports and no known neurovascular, inflammatory, or neurodegenerative diseases. The recruitment-related biases are discussed in the Discussion section. All subjects underwent clinical, neuropsychological, and neuroimaging assessments on the same day. For patients, these assessments were conducted at least 3 months post-stroke (median: 501 days, min: 91, max: 2674 days). Informed consent was obtained from all participants. Symptoms at onset were categorized as cognitive, motor/vertebrobasilar, or a combination of both. Motor/vertebrobasilar symptoms included vertigo, visual disturbances, ataxia, dysarthria, sensory deficits, dysphagia, weakness, and other issues related to movement and muscle control. Since certain symptoms, such as ataxia and weakness, overlap between motor and vertebrobasilar categories, they were grouped together into a single category for simplicity. Non-inclusion criteria were known neurovascular, inflammatory, or neurodegenerative diseases.

### MRI acquisition

3D T1-MPRAGE sequences were acquired on a 3 T scanner (Philips Achieva). For the first study, the following parameters were: 1*1*1 mm voxel size, flip angle = 8°, FOV = 240*240, slice number = 170; and for the second study: 0.9*0.9*1 mm voxel size, flip angle = 8˚, FOV = 256 × 256, slice number = 189.

### Neuropsychological assessment

The following tests were used: the Free and Cued Selective Reminding test [16] (verbal anterograde memory); the DMS48 task [17] (visual anterograde recognition memory); the Stroop test [18] (inhibition); literal and semantic fluencies [18] (executive functions); D2 [19] (attention); digit-symbol test [20] (working memory); ExaDé confrontation naming test [21] (language), visuospatial and auditory-verbal digit span [20] (working memory) and three mood and affective scales: the State-Trait Anxiety Inventory [22], Starkstein Apathy Scale [23] and Beck Depression Inventory Scale [24].

### Neuropsychological analyses

Group comparisons were carried out using a χ^2^ test for nominal data. Neuropsychological test scores were standardized to z-scores using normative scales. The psycho-affective scales were analyzed using raw data due to a lack of adequate normative scales. To minimize multiple comparisons, one subtest by neuropsychological test was selected, and the mean z-score by cognitive function was computed: working memory (auditory-verbal and visuospatial digit span), verbal memory (FCSRT Delayed Free Recall), executive function (digit-symbol, Stroop interference minus denomination (I−D) response time), language (literal and semantic fluencies, confrontation naming test) and attention (D2 GZ-F, number of processed items minus errors). A mean score of these cognitive functions was calculated to reflect general cognitive performance, referred to as “Total.”

#### Cluster analysis

To identify subgroups of patients with similar performances independently of infarct laterality, a cluster analysis was performed using the k-means method and Euclidean distance in both Rstudio and Python [25]. Our data set Hopkins clustering tendency was 0.68 (0: uniformly distributed; 0.5: random; 1: highly clustered data). One patient lacking a value in the attention function was excluded. To identify factors and variables that could better explain the clustering, a logistic regression using the stepwise method on JASP was computed.

#### Principal component analysis (PCA)

To further explore how patients and healthy subjects could be differentiated by their cognitive performances, a PCA using the selected cognitive functions in Rstudio was performed. Results are represented by a plot of each individual projected into the space of the two principal components (PC), explaining the maximal amount of variance in the dataset. Individuals are colored by clusters. The percentage of explained variance by PC and the percentages of the contribution of each variable to those PC are represented in Supplementary Fig. 1.

#### rmANOVA and boxplots

To compare healthy subjects and patients with left, right, and bilateral lesions, a repeated measure ANOVA was conducted using the z-scores by cognitive functions. In the event of a grouping effect, we employed a post hoc t-test. Finally, we used a Kruskal–Wallis test followed by Bonferroni-corrected Dunn’s tests to quantify the most discriminative test and study the “Total” cognitive function. Significant results were represented as boxplots using z-scores by groups in order to analyze individual performances.

### Neuroimaging analysis

#### Lesion volumetry and location

Lesions were manually segmented on the native T1w images by two independent investigators using MRIcron, and the resulting overlapping mask was used (mean dice = 0.84).

Lesions were localized using HIPS-THOMAS (Histogram-based Polynomial Synthesis-Thalamus Optimized Multi-Atlas Segmentation; [26, 27]). Nuclei were gathered into nuclear groups using Morel’s atlas repartition (Anterior: AV, AM, AD, LD; Medial: Hb, MD, MV, Pv, CeM, CL, CM, Pf, sPf; Posterior: LGN, Li, LP, SG, MGN, Po, PuA, PuI, puL, PuM; Lateral: VA, VL, VM, VPI, VPL, VPM) [28, 29]. The mean percentage of lesions affecting each nuclear group by cluster was computed. If a lesion reached the MTT with a volume greater than 5 mm^3^, the MTT was considered to be disrupted using Morel’s segmentation to avoid any lesion bias on the tract reconstruction and, thus, disruption identification. The presence, absence of an IA or thalamic lesion extending into it was determined by two independent raters following a previously established protocol [14].

#### Normalization

Native T1w images and segmented lesions were registered to the MNI152 template using an affine and diffeomorphic deformation for bilateral lesions and enantiomorphic transformation for unilateral lesions after skull stripping using the BCBtoolkit [30]. Normalized overlapping lesions of all patients and by clusters are represented on the MNI152 template using MRIcroGL.

#### Disconnectome

To map the impact of thalamic strokes on white matter tracts, we used the Disconnectome maps software of the BCBtoolkit [31]. The software output is, for each patient, a map associated with voxel-wise probabilities of disconnection within the standard MNI152 space. The probability that each tract might be disconnected was given by the Tractotron software from the same toolkit. Tracts with a disconnection probability above 50% were considered to be significant [31] and used as a threshold for the disconnectome maps. An average disconnectome map of all patients by clusters by laterality of infarct was projected onto an MNI152 template to identify commonly affected tracts.

#### Cortical thickness

The T1w images were processed using FreeSurfer (http://surfer.nmr.mgh.harvard.edu) to obtain surface parcellation based on the Desikan-Killiany atlas for each subject. The mean cortical thickness of the whole brain and specific cortical areas found to be connected to the most disrupted tracts identified by the previous analyses were compared between groups using a Mann–Whitney *U* test.

## Results

### Sociodemographic

**Fig. 1:**
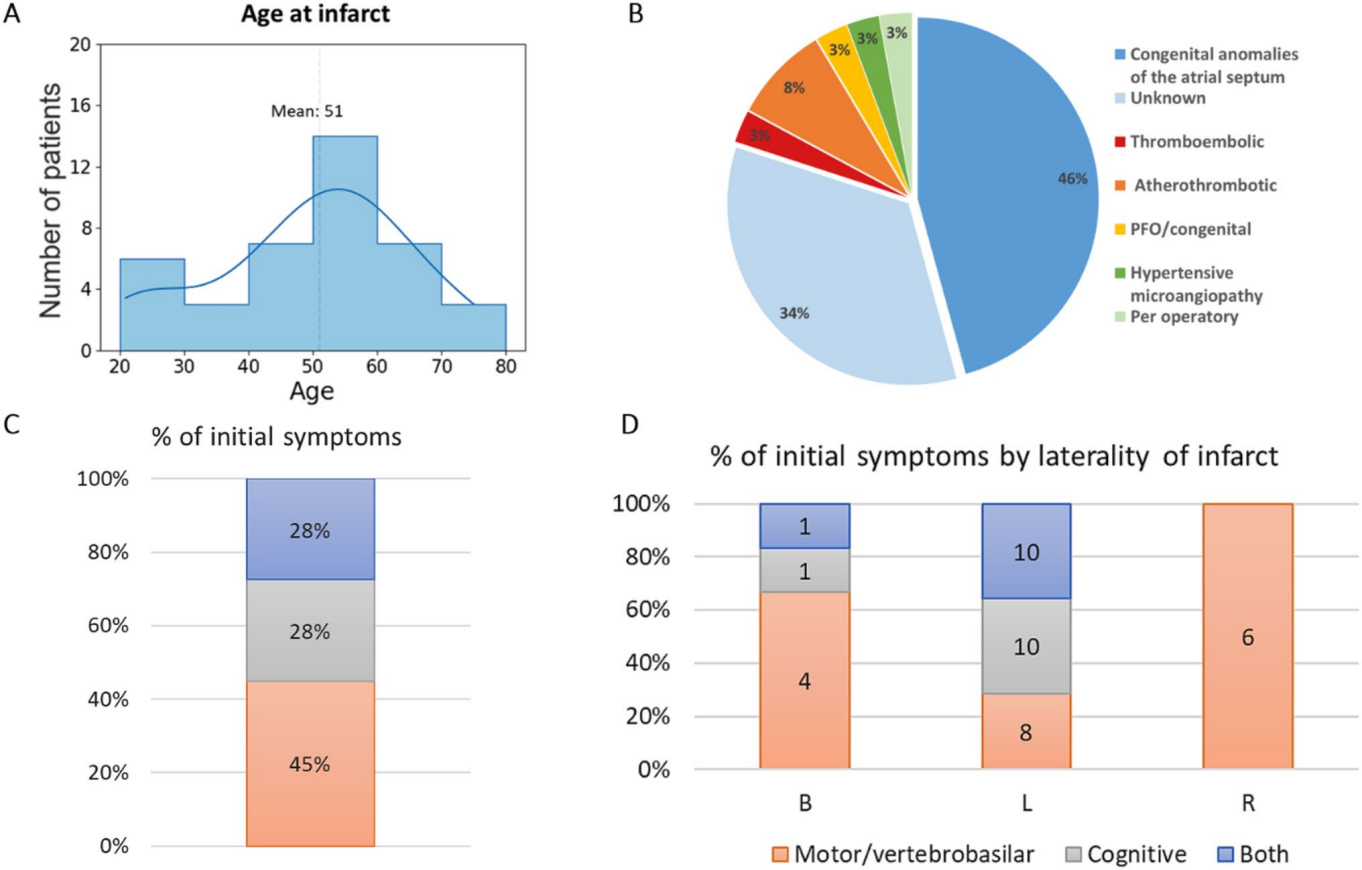
**A** Distribution of patient ages at the time of infarct. **B** Percentages of thalamic stroke etiologies, including congenital atrial septum anomalies like atrial septal defect ostium secondum, Patent Foramen Ovale (PFO), and/or atrial septal aneurysm (data missing for five patients). **C** Percentages of patients by categories of initial stroke symptoms. **D** Symptom prevalence comparison based on infarct laterality: B (Bilateral), L (Left), R (Right)

Groups, comprising 45 healthy subjects and 40 patients with ischemic thalamic stroke were comparable in terms of gender (χ^2^ = 2.78, *p* = 0.10), age (*t*-test, *p* = 0.41), and years of education (*t*-test, *p* = 0.12) (Table 1). The mean age at stroke was 51 with a minimum of 23 and a maximum of 75 (Fig. 1A). The origin of the thalamic stroke was in almost half of the cases (46%) a congenital anomaly of the atrial septum (Fig. 1B). Initial symptoms at onset were equally divided between motor/vertebrobasilar (28%), cognitive (28%) and both (45%) (Multinomial test: χ^2^ = 2.45; *p* = 0.3) (Fig. 1C). However, right thalamic strokes did not lead to cognitive symptoms at the acute phase, contrary to left or bilateral strokes (Fig. 1D) (χ^2^ = 11.5; *p* = 0.02).

**Table 1:** Sociodemographic information from the dataset

	Healthy subjects	Patients
N	45	40
Gender (F)	25	15
Lesion side (left–right-bilateral)	–	28–6-6
Mean age ± SD (min–max)	48.5 ± 13.7 (20–69)	51.1 ± 14.9 (23–75)
Mean years of education ± SD (min–max)	13.58 ± 3.5 (5–21)	12.4 ± 3.4 (4–17)

### Neuropsychological analyses

To identify subgroups of patients and compare them with healthy control subjects, a cluster analysis including all patients and control subjects was conducted using the k-means method on verbal memory, attention, working memory, executive functions, and language functions. A two-cluster solution was optimal for portioning the data, obtaining 13/30 indices from the NbClust R package (the second-best option was three clusters, giving 6/30 indices). Cluster 1 included 96% of healthy subjects and 51% of patients (*n* = 20), while Cluster 2 consisted of the remaining patients (*n* = 19) and two healthy subjects (χ^2^ = 22; p < 0.001, comparing the repartition of healthy subjects and patients across clusters).

**Fig. 2:**
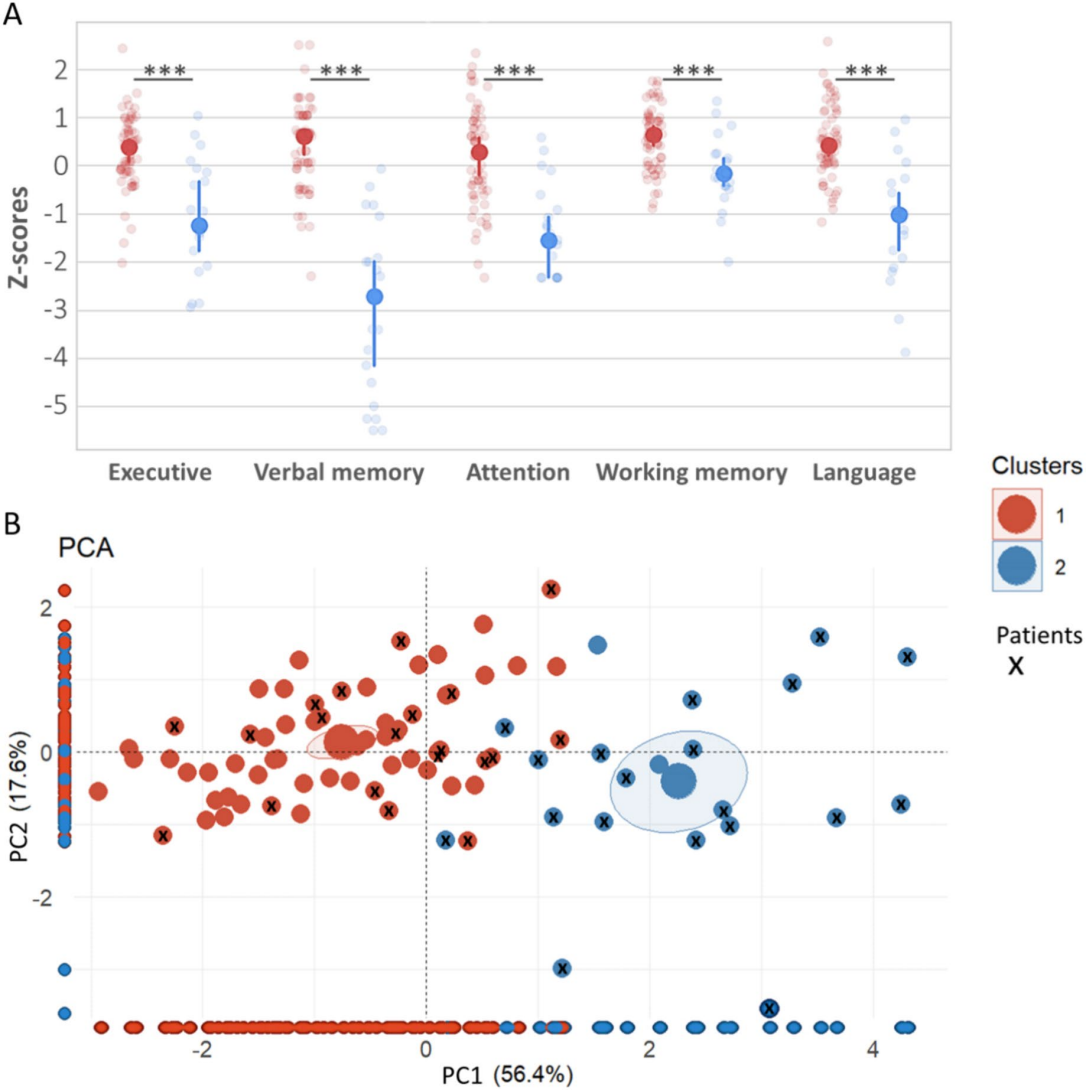
**A** Comparison of neuropsychological test z-scores between patients and healthy subjects across both clusters. Solid dots show the median, bars indicate 95% confidence intervals, and each dot represents an individual's performance. Clusters differ significantly across all cognitive functions (Mann–Whitney, *p* < 0.001). **B** PCA plot: Larger dots represent cluster means with 95% confidence intervals. Subjects are color-coded by cluster (from panel **A**), and patient dots have an “x” (only in panel B for clarity). Individuals are projected on the x or y axes, showing how each component distinguishes the clusters

Patients in Cluster 1 exhibited no or minor cognitive deficits (z-scores around 0) and were indistinguishable from healthy subjects, as they were clustered together (Fig. 2A).

Several factors differed between the clusters (Table 2). Cluster 2 patients were older (55.6 vs 47.6 years old, Mann–Whitney, *p* = 0.025), had fewer years of education (Mann–Whitney, *p* = 0.002), and larger volume of lesions (Mann–Whitney, *p* < 0.001; mean ± SD (mm^3^): Cluster 1 = 229 ± 214; Cluster 2 = 553 ± 289). Infarct laterality was also significantly different between clusters (χ^2^ = 12; *p* = 0.002): Cluster 1 included all patients with right lesions, while Cluster 2 contained all with bilateral lesions. Likewise, subjects were unequally distributed based on the presence of, absence of, or damage to the IA (*χ*^2^ = 8.4; *p* = 0.015). In Cluster 1, 76% of subjects had an IA, and among them, patients with damage extending into it represented 10% of the dataset, while in Cluster 2, 67% had an IA, and patients with a damaged IA represented 43% of the sample. Patients with left lesions were evenly distributed between clusters. Importantly, Cluster 2 contained 100% of the patients with an interrupted MTT (Table 2). In addition, the time between the infarct, neuroimaging, and neuropsychological assessment did not differ between clusters (*T*-test, *p* = 0.74), and infarct etiologies were also similar (*χ*^2^ = 8.3; *p* = 0.22).

**Table 2:** Comparison of subjects from both clusters, with lesion volume calculated in MNI space. Two healthy subjects could not be evaluated for the IA due to kissing thalami

	Cluster 1	Cluster 2
N total subjects /healthy subjects/patients	63-43-20	21–2–19
Gender (F)	34	6
Mean age ± sd	47.6 ± 13.9	55.6 ± 14.1
Mean years of study ± sd	13.7 ± 3.2	10.8 ± 3.4
Laterality Left-Right-Bilateral	14-6-0	13-0-6
Interthalamic adhesion (Yes/No/Damaged)	43-13-5	8-7-6
Mamillothalamic tract lesion	0	9
Mean lesion volume (mm)^3^ ± sd	229 ± 214	553 ± 289
Days since infarct ± sd	583 ± 630	650 ± 612

A PCA was performed to explore how cognitive functions differentiate patients from healthy subjects. The first PC (PC1) explained 56.4% of the variance, while the second (PC2) explained 17.6%. In PC1, significant contributions came from attention (27%), executive functions (27%), and verbal memory (20%), while PC2 was influenced by language (42%) and working memory (34%) (Supp. Figure 1). The projection of individual performances and cluster attribution into a two-dimensional space (Fig. 2B) showed that PC1 nearly perfectly distinguished patients from Cluster 1 and Cluster 2, indicating that associated variables were more discriminative and impaired in patients from Cluster 2. This confirmed the absence or minor deficits in patients from Cluster 1 (marked as “x” on red dots, Fig. 2B). Only two patients were slightly closer to subjects from Cluster 2 on PC1, but were also close to the two healthy subjects in that group (absence of “x” on blue dots, Fig. 2B).

A logistic regression analysis was performed to identify factors predicting Cluster membership (Table 2). The most predictive factor for Cluster 2 was MTT interruption (χ^2^ = 16, *p* < 0.001, odds ratio = 4.3 × 10⁻⁹). This model achieved an accuracy of 0.95 and an area under the curve of 0.98. The second-best model also included infarct laterality (χ^2^ = 5, *p* < 0.001). When cognitive functions were incorporated, the model considering only verbal memory showed the highest predictive power (*χ*^2^ = 36, *p* < 0.001), with perfect accuracy and specificity for predicting cluster membership.

**Fig. 3:**
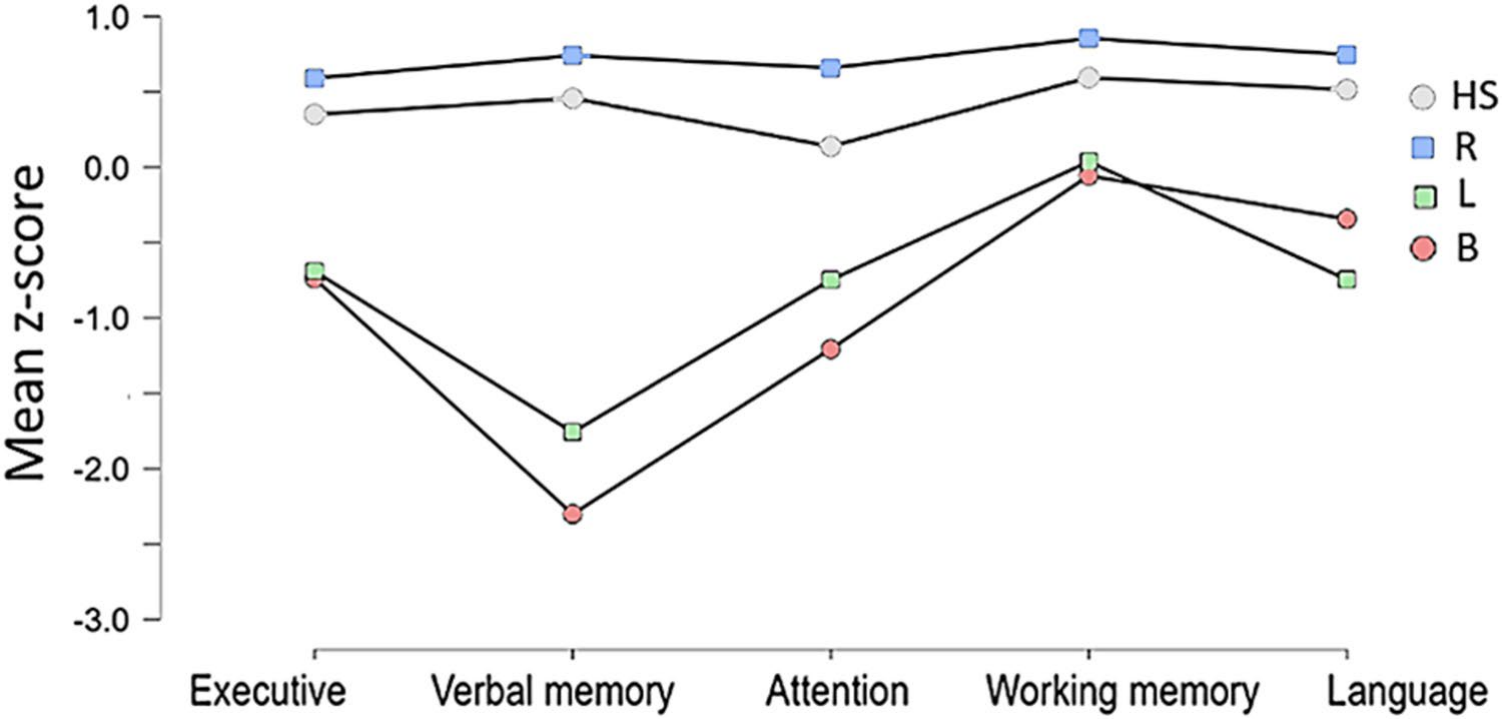
Mean z-score in each cognitive function by groups of patients depending on the laterality of their infarct

Given that infarct laterality appears to be a key factor in clustering, a repeated measures ANOVA (rmANOVA) was conducted on the five cognitive functions with infarct laterality as the grouping factor. This analysis revealed a main effect of infarct laterality (*p* < 0.001, ηp^2^ = 0.47). Post hoc comparisons showed that patients with bilateral or left strokes were significantly more impaired than both healthy subjects (*p* < 0.001) and those with right thalamic strokes (*p* < 0.001). Results for each cognitive function are detailed in Fig. 3, with individual boxplots presented separately in Supplementary Fig. 2.

No differences on the psychoaffective scales (Beck, Starkstein, Spielberg; Mann–Whitney, *p* > 0.3) were demonstrated between healthy subjects and patients based on infarct laterality (Mann–Whitney, *p* > 0.3). However, Starkstein apathy scores were significantly higher in Cluster 2 than in Cluster 1 (Mann–Whitney, *p* = 0.003), a finding that remained significant when comparing only patients from both clusters (*p* = 0.01; mean ± SD for Cluster 1: 7.8 ± 4.5; Cluster 2: 11.4 ± 4.2).

### Neuroimaging analyses

**Fig. 4:**
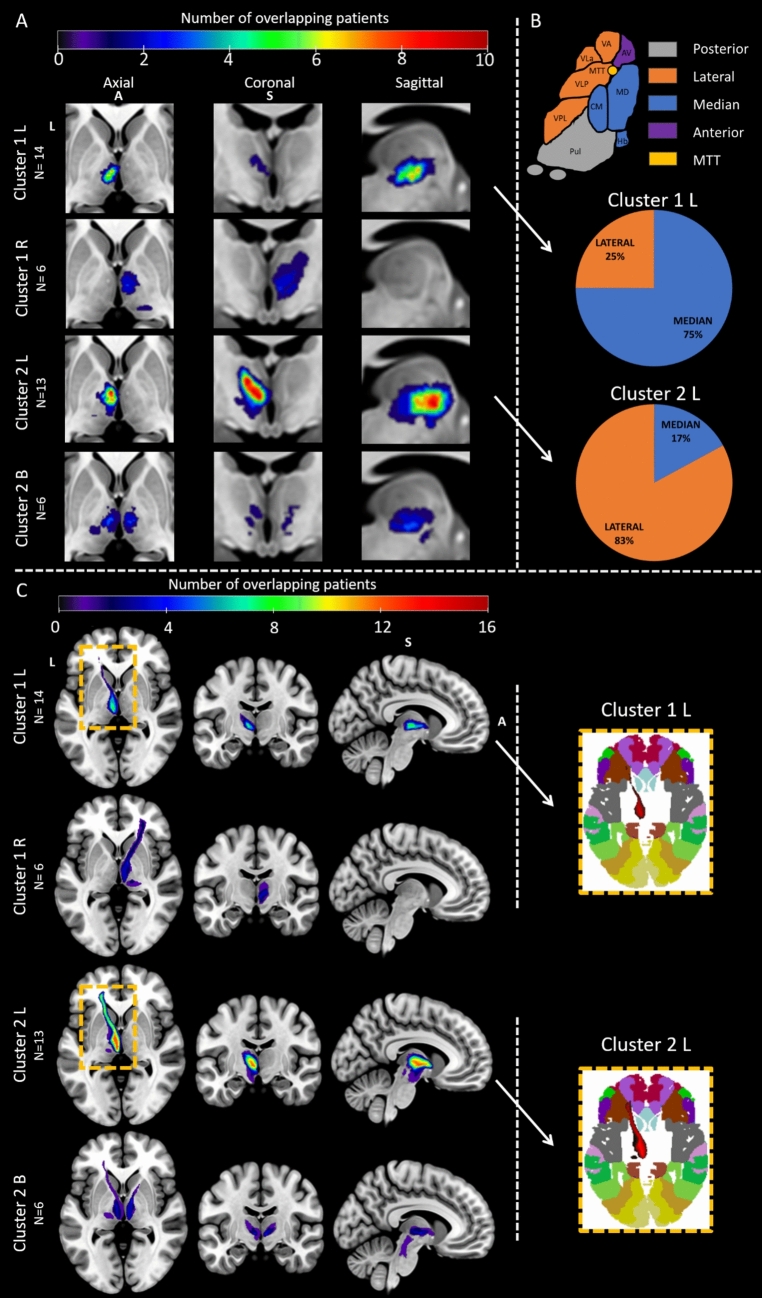
**A** Overlapping normalized lesions on an MNI152 slice showing the most affected voxels of Cluster 1 patients with left (*n* = 14) or right (*n* = 6) lesions, and Cluster 2 patients with left (*n* = 13) or bilateral lesions (*n* = 6). Cluster 1's left infarcts primarily affected the median thalamus, while Cluster 2's were mainly lateral. **B** Nuclei distribution (top) and percentage of lesioned nuclear groups for left-lesioned patients from both Clusters, segmented with HIPS-THOMAS (bottom). **C** Mean disconnectome maps by Cluster and infarct laterality, along with Brodmann atlas projections in the MNI152 space (right inset). Left anterior thalamic radiations were the most commonly disconnected tract, with Cluster 2's left infarcts showing stronger disconnection. When mapped onto the Brodmann atlas, these disrupted radiations projected to areas 11 and 47, linked to the orbitofrontal cortex and inferior frontal gyrus. *A* anterior, *S* superior, *L* left. Color bars show the number of patients with overlapping lesioned voxels

A puzzling issue arose with patients with a left thalamic infarct, as they were evenly distributed between Cluster 1 (*n* = 14) and Cluster 2 (*n* = 13). This raises the question of why the same infarct laterality could result in minimal cognitive impact for some patients but significant effects for others? To investigate, neuroimaging analyses were performed. Overlapping lesions, categorized by clusters and infarct laterality, are displayed on the MNI152 template (Fig. 4A). Most lesions were in the median and lateral left thalamic territories (Fig. 4B). In Cluster 1 patients with left infarcts, 75% affected the median thalamus, while 25% impacted the lateral part. Conversely, Cluster 2 patients with left infarcts showed the opposite pattern (17% vs 83%).

To assess both local and distant effects of lesions, a disconnectome map was generated for each patient [31]. Thresholded at 50% probability, this map isolated voxels representing tracts with a high likelihood of disruption. Average disconnectomes for each cluster, based on infarct laterality, are shown in Fig. 4C. The most disconnected tract was the left anterior thalamic radiations, affecting 17/19 patients from Cluster 2 and 13/20 from Cluster 1. Patients with left infarct in Cluster 2 exhibited more pronounced left anterior thalamic disconnection (right inset in Fig. 4C). When overlapped to the Broadmann atlas, these disrupted radiations projected to areas 11 and 47, corresponding to the orbitofrontal cortex and the inferior frontal gyrus.

Given the high likelihood of lesions bilaterally interrupting anterior thalamic projections to the orbitofrontal and ventrolateral prefrontal cortex (vlPFC), their mean cortical thickness was assessed in both hemispheres, along with the anterior cingulate gyrus and dorsolateral prefrontal cortex (dlPFC) and the whole brain. Indeed, these regions are demonstrated to be connected to the anterior and mediodorsal nuclei of the thalamus via the anterior thalamic radiations [32, 33]. This analysis included control subjects due to the small patient group size. Cluster 2 subjects showed significantly reduced mean cortical thickness in the whole brain and in each cortical area of interest (*p* < 0.005) (Table 3).

**Table 3:** Mean cortical thickness for the whole brain and by cortical parcellations between Clusters 1 (*n* = 63) and 2 (*n* = 21) using a Mann–Whitney *U* test

	Cluster	Mean	SD	*P*-value
Whole brain	1	2.40	0.1	0.004
	2	2.32	0.09	
Orbitofrontal left	1	2.41	0.11	0.02
	2	2.35	0.11	
Orbitofrontal right	1	2.45	0.11	0.055
	2	2.39	0.10	
dlPFC left	1	2.42	0.12	0.005
	2	2.33	0.11	
dlPFC right	1	2.42	0.12	0.2
	2	2.38	0.14	
vlPFC left	1	2.47	0.15	0.01
	2	2.38	0.13	
vlPFC right	1	2.47	0.13	0.05
	2	2.40	0.13	
Cingular left	1	2.34	0.11	0.002
	2	2.25	0.11	
Cingular right	1	2.45	0.15	0.70
	2	2.47	0.22	

Significant *p*-values are underlined

## Discussion

This study aimed to explore the neuropsychological outcomes of ischemic thalamic strokes, particularly focusing on factors influencing poor cognitive recovery. Our findings suggest that cognitive impairments following thalamic strokes might not be as uniform or as severe as previously thought. Our cluster analysis identified one subgroup of patients (Cluster 1) with no or minimal cognitive impairment, consistent with previous studies [8–13]. This subgroup included all patients with right thalamic lesions and about half of those with left thalamic infarcts. This finding is notable as it suggests effective compensatory or neuroplastic mechanisms that may prevent significant functional deficits after isolated thalamic lesions.

The other subgroup of patients (Cluster 2) exhibited significant cognitive impairments across several domains, particularly in verbal memory, attention, and executive functions, and a higher apathy score. This subgroup mainly consisted of patients with left thalamic damage (49% of patients with a left infarct) and all patients with bilateral lesions. This aligns with previous studies indicating that left or bilateral thalamic strokes result in more severe cognitive deficits compared to right-sided lesions [13, 34]. A novel aspect of our study is the observation that, following a left thalamic infarct, approximately half of the patients recover well, while the other half show significant cognitive deficits.

A major aim of the current study was to analyze the factors that could explain poor outcomes following a thalamus stroke. We identified that, in addition to the laterality effect, subjects from Cluster 2 were older and had a lower level of education. Cluster 2 patients also had larger lesion volumes, and all identified MTT disruptions, which consistently resulted in more severe cognitive impairments. Additionally, the absence of an IA or lesions extending into it was linked to poorer outcomes.

The intricate relationship between lesion location within the thalamus and resultant cognitive deficits underpins the complexity of thalamic contributions to cognitive functions. Our neuroimaging analysis revealed that lesions predominantly affecting the left thalamus, especially those involving the lateral regions, were associated with more severe cognitive impairments, especially in verbal memory, attention, and executive functions (Cluster 2). Similar findings have been reported for the anterior left thalamic region, linked to mnesic deficits [2, 12, 34] or severe impairments in language and executive functions following stroke [13]. Hwang et al. [34] identified a comparable region, suggesting a distinction between connector hubs and provincial hubs within the thalamus. Indeed, connector hubs, with their extensive connectivity to multiple cortical networks, may play a critical role in cognitive outcomes. Additionally, the MTT, a crucial tract involved in memory, is located in the same region associated with severe cognitive impairments [2, 12]. Altogether, the relatively small size of this critical area associated with a low likelihood of being affected may explain why many stroke patients reported in recent studies remain relatively cognitively preserved. In contrast, lesions in this left lateral area, particularly those involving the MTT, are more likely to result in pronounced cognitive deficits.

The anterior thalamic radiations connect the anterior and mediodorsal nuclei of the thalamus with the dlPFC, vlPFC, OFC, and anterior cingulate gyrus [32]. The dlPFC is associated with working memory, vlPFC with executive functions, OFC with emotional control and goal-directed behavior while the anterior cingulate gyrus is part of the Papez circuitry for mnesic functions [33]. The cortical thickness of subjects from Cluster 2 was decreased in the left OFC, dlPFC, vlPFC and anterior cingulate gyrus; this may be related to the more frequently interrupted anterior thalamic radiations. This implies that thalamic strokes may have far-reaching implications beyond the immediate site of the lesion in some patients in accordance with the hub hypothesis.

Our study has some limitations. It is worth reminding that the thalamic stroke patients in our dataset are relatively young (average age of 51 years) compared to typical stroke populations. In addition, a significant percentage of strokes were attributed to congenital anomalies of the atrial septum, such as patent foramen ovale (PFO), which increase the risk of cardioembolism. However, a meta-analysis has indicated that PFO is present in 10 to 44% of stroke cases, particularly among individuals aged 55 and younger [35]. This finding aligns with the relatively young age of our cohort. Lesioned nuclei supplied by more susceptible arteries (i.e., paramedian) were overrepresented in our cohort, which is also due to inclusion criteria from our second study on dorsomedian nuclei lesions. Future research should include a broader range of affected arterial territories. Nevertheless, our study is notable for its wide age range (18–80 years) and large cohort of patients with chronic isolated thalamic infarcts, with neuropsychological assessments surpassing those of many previous studies (e.g., [12]: 22 patients; [10]: 17 patients; [9]: 19 patients; [8]: 68 patients, [36]: 20 patients). While our findings contribute significantly to the literature, broader generalizability may be better achieved in future research.

To conclude, our findings particularly challenge the uniformity and severity of cognitive impairments traditionally associated with thalamic lesions by demonstrating that most patients had no or minor cognitive deficits. Our analysis also revealed the heterogeneity in cognitive outcomes depending on the lesion’s laterality and the specific thalamic territories impacted. Notably, lesions within the left thalamus and those affecting the mamillothalamic tract significantly correlate with more profound cognitive deficits, particularly in verbal memory, attention, and executive functions. In addition, our connectome analysis points to effects far beyond the thalamus proper of thalamic strokes. These results suggest that we should adopt a more holistic understanding of the thalamus when considering its integral role in the broader neural network governing cognitive functions.

## Supplementary Information

Below is the link to the electronic supplementary material.

Supplementary file1 (DOCX 686 KB)

## Data Availability

Datasets are available from the corresponding author upon reasonable request.
